# Investigations into the Antibacterial Activity of the Silver-Based Antibiotic Drug Candidate SBC3

**DOI:** 10.3390/antibiotics1010025

**Published:** 2012-11-20

**Authors:** Michael A. Sharkey, James P. O’Gara, Stephen V. Gordon, Frauke Hackenberg, Claire Healy, Francesca Paradisi, Siddappa Patil, Bettina Schaible, Matthias Tacke

**Affiliations:** 1 UCD Conway Institute of Biomolecular and Biomedical Research, University College Dublin, Belfield, Dublin 4, Ireland; Email: michael.sharkey@ucd.ie (M.A.S.); jim.ogara@ucd.ie (J.P.O.); stephen.gordon@ucd.ie (S.V.G.); claire.healy@ucdconnect.ie (C.H.); francesca.paradisi@ucd.ie (F.P.); bettina.schaible@googlemail.com (B.S.); 2 UCD School of Chemistry and Chemical Biology, University College Dublin, Belfield, Dublin 4, Ireland; Email: frauke.hackenberg@ucdconnect.ie (F.H.); patilsiddappa@gmail.com (S.P.)

**Keywords:** silver, *N*-heterocyclic carbene, MRSA

## Abstract

The synthesis of *N*-heterocyclic carbene (NHC) silver(I) acetate complexes with varying lipophilic benzyl-substituents at the 1 and 3 positions starting from 4,5-diphenylimidazole, opened a new class of antibiotic drug candidates. These NHC-silver(I) acetate derivatives exhibit interesting structural motifs in the solid state and proved to be soluble and stable in biological media. The leading candidate, SBC3, which was known to exhibit good antibacterial activity in preliminary Kirby-Bauer tests, was tested quantitatively using minimum inhibitory concentrations. NHC-silver(I) acetate complexes were found to have MIC values ranging from 20 to 3.13 μg/mL for a variety of Gram-positive, Gram-negative and mycobacteria tested. These values represent good antibiotic activities against potential pathogens when compared to clinically approved antibiotics. Most striking is the fact that SBC3 is active against methicillin-resistant *Staphylococcus aureus* with a MIC value of 12.5 μg/mL.

## 1. Introduction

*Staphylococcus aureus* (*S. aureus* or SA) is a common type of bacteria found in about 30 percent of healthy people, primarily on the skin or in the nose. Most of these individuals are healthy, but some people become infected with SA indicating that the bacteria can penetrate through a break in the skin causing skin and tissue infections. SA can also cause more serious illnesses such as surgical wound infections, bloodstream infections, bone infections, and pneumonia typically in hospital settings. In the past few decades, a more dangerous form of SA has emerged, which is known as methicillin-resistant *S. aureus* (MRSA). What sets MRSA apart is that it is resistant to the entire class of commonly prescribed β-lactam antibiotics including methicillin, amoxicillin and oxacillin among others; an effect firstly documented in 1964 [1]. In the meantime MRSA has grown to a major health problem leading to significant mortality worldwide [2]. The same is true for *Mycobacterium tuberculosis*, the causative agent of TB in humans, which is developing increasing resistance to front-line antibiotics [3], demanding that new lead compounds are urgently needed for the drug development pipeline. 

**Figure 1:**
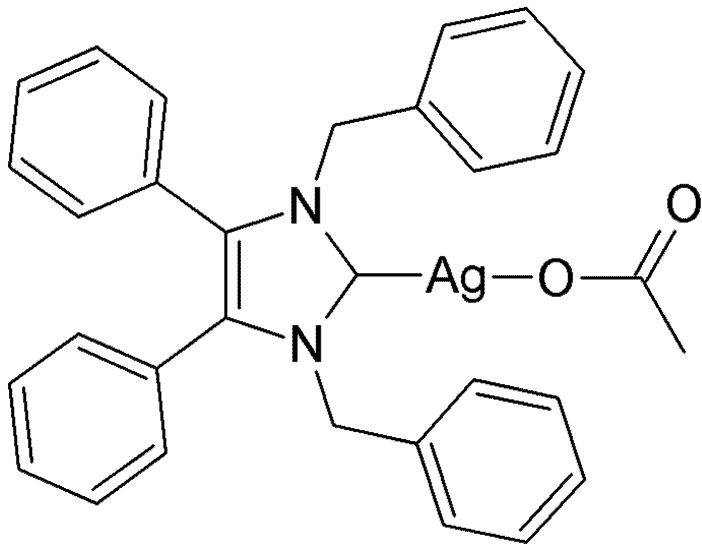
Molecular structure of SBC3.

This emergence of resistant bacteria has triggered interest from industry and academia to synthesise and evaluate new antibiotic drug candidates like carbene-silver acetates derived from *N*-heterocyclic (NHC) carbenes [4,5,6]. The lead compound from our group (1,3-dibenzyl-4,5-diphenylimidazol-2-ylidene) silver(I) acetate (SBC3) showed strong inhibition of *E. coli* and *S. aureus* in preliminary Kirby-Bauer tests [7] and its molecular structure is shown in Figure 1. 

SBC3 is therefore investigated in this paper for its antibacterial activity against a variety of bacterial strains using minimum inhibitory concentration (MIC) tests. 

## 2. Results and Discussion

For the antibiotic testing of SBC3 the following seven bacterial strains were chosen: *Mycobacterium bovis BCG Pasteur*, *Mycobacterium smegmatis*, *Salmonella typhimurium*, *Staphylococcus aureus* BH1CC, a methicillin-resistant *S. aureus (*MRSA) strain, BH1CC ΔSCC*mec*, an isogenic methicillin sensitive (MSSA) derivative of BH1CC, *Pseudomonas aeruginosa* and *Escherichia coli* (NCIB strain 9485). The results of the minimum inhibitory concentration testing of SBC3 against the full bacterial panel after an incubation period of 20 h delivered MIC values between 20 and 3.13 μg/mL; the complete experimental results are shown in Table 1.

**Table 1:** Minimum inhibitory concentration (MIC) of SBC3 against different bacterial strains.

Bacterial Strain	MIC [μg/mL]
*Mycobacterium bovis BCG Pasteur*	20
*Mycobacterium smegmatis*	5
*Salmonella typhimurium*	12.5
*Staphylococcus aureus (MSSA)*	12.5
*Staphylococcus aureus (MRSA)*	12.5
*Pseudomonas aeruginosa*	3.13
*Escherichia coli*	6.25

SBC3 shows significant activity against the *M. bovis BCG Pasteur* and *M.smegmatis* with MIC values of 20 and 5 μg/mL. Similar activities resulting in MIC values of 12.5 μg/mL are also found for *Salmonella typhimurium* and *S. aureus*; the latter bacterium is inhibited at the same concentration independent of its methicillin susceptibility. The best activities of SBC3 are found against *E. coli* and *P. aeruginosa* with MIC values of 6.25 and 3.13 μg/mL.

This MIC value of around 3 μg/mL for SBC3 with a molecular weight of 567 g/mol is already quite impressive since conventional antibiotics like Imipenem (β-Lactam Antibiotic/Carbapenem, 299 g/mol), Ceftazidime (β-Lactam Antibiotic/Cephalosporin, 547 g/mol) and Piperacillin + Tazobactam (β-Lactam Antibiotic, 518 g/mol) inhibit at the same concentrations of 3–6 μg/mL against the pathogen *P. aeruginosa*.

## 3. Experimental

The following bacterial strains were used for the antibiotic testing: *Mycobacterium bovis BCG Pasteur*, *Mycobacterium smegmatis*, *Salmonella typhimurium*, *Staphylococcus aureus* BH1CC, a methicillin-resistant *S. aureus (*MRSA) strain, BH1CC ΔSCC*mec*, an isogenic methicillin sensitive (MSSA) derivative of BH1CC, *Pseudomonas aeruginosa* and *Escherichia coli* (NCIB strain 9485).

25 mg of the compound SBC3 was dissolved in 2.5 mL of 100% DMSO, giving a concentration of 10 mg/mL in each case. In order to progress, 0.02 mL of this primary solution was added to 1.98 mL Mueller Hinton II broth (MHBII) and 0.2 mL placed in wells in the first row of a microtitre plate. The concentration of SBC3 is 100 μg/mL in row 1 wells containing it at this stage. 0.1 mL MHBII was placed in all other wells of the microtitre plate and 0.1 mL of the material in row 1 was transferred to wells in the next row (already containing 0.1 mL of MHBII broth), where mixing took place by repeatedly pipetting up and down, and this was continued down the plate so that a dilution series was set up. Each well was inoculated with 0.1 mL of MHBII containing ~1 × 10^5^ bacterial cells, and the plates incubated overnight at 37 °C. With the addition of the inoculum, the concentration of SBC3 in the first row became 50 μg/mL; for the two *Mycobacteria* an analogues protocol was used and the highest concentration of SBC3 was 40 μg/mL. Tests were performed in duplicate for each strain, and one DMSO control without SBC3 for each strain was included in the test. After an incubation period of 20 h, the absorbance of the wells was read using a plate reader set to measure at 600 nm. An OD600 absorbance reading <0.1 was interpreted as no growth of the bacterial strain; the plates were examined visually for growth as well.

## 4. Conclusions

SBC3 is a low molecular weight and lipophilic drug-like molecule, which combines high stability and low light-sensitivity in the solid state with good stability and antibacterial activity in the biological medium. It shows significant activity against the *M. bovis BCG Pasteur* and *M.smegmatis* as well as against *Salmonella typhimurium*, MSSA and MRSA. The best activities are found for *E. coli* and *P. aeruginosa*, which makes SBC3 already as active as conventional β-lactam antibiotics against these two bacterial strains. Breaking the resistance in MRSA is also a good argument for the further development of silver-based antibiotic drug candidates like SBC3.

## References

[1] Sutherland R., Rolinson G.N. (1964). Characteristics of methicillin-resistant staphylococci. J. Bacteriol..

[2] Bartlett J.G. (2008). Methicillin-resistant *Staphylococcus aureus* infections. Top. HIV Med..

[3] Lienhardt C., Raviglione M., Spigelman M., Hafner R., Jaramillo E., Hoelscher M., Zumla A., Gheuens J. (2012). New drugs for the treatment of tuberculosis: needs, challenges, promise, and prospects for the future. J. Infect. Dis..

[4] Hindi K.M., Panzner M.J., Tessier C.A., Cannon C.L., Youngs W.J. (2009). The Medicinal Applications of Imidazolium Carbene Metal Complexes. Chem. Rev..

[5] Panzner M.J., Deeraksa A., Smith A., Wright B.D., Hindi K.M., Kascatan-Nebioglu A., Torres A.G., Judy B.M., Hovis C.E., Hilliard J.K. (2009). Synthesis and in vitro Efficacy Studies of Silver Carbene Complexes on Biosafety Level 3 Bacteria. Eur. J. Inorg. Chem..

[6] Patil S., Tacke M., Melník M., Segľa P., Tatarko M. (2011). NHC-Silver(I) Acetates as Bioorganometallic Anticancer and Antibacterial Drugs. Insights into Coordination, Bioinorganic and Applied Inorganic Chemistry.

[7] Patil S., Deally A., Gleeson B., Müller-Bunz H., Paradisi F., Tacke M. (2011). Novel Benzyl-Substituted *N-*Heterocyclic Carbene-Silver Acetate Complexes: Synthesis, Cytotoxicity and Antibacterial Studies. Metallomics.

